# Bacteriophages: The promising therapeutic approach for enhancing ciprofloxacin efficacy against bacterial infection

**DOI:** 10.1002/jcla.24932

**Published:** 2023-06-28

**Authors:** Aref Shariati, Milad Noei, Zahra Chegini

**Affiliations:** ^1^ Molecular and Medicine Research Centre Khomein University of Medical Sciences Khomein Iran; ^2^ Department of Genetics, Faculty of Advanced Science and Technology, Tehran Medical Sciences Islamic Azad University Tehran Iran; ^3^ Department of Microbiology, School of Medicine Hamadan University of Medical Sciences Hamadan Iran

**Keywords:** antibiotic resistance, bacterial biofilm, bacteriophages, ciprofloxacin, combination therapy

## Abstract

**Background:**

The emergence of ciprofloxacin‐resistant bacteria is a serious challenge worldwide, bringing the need to find new approaches to manage this bacterium. Bacteriophages (phages) have been shown inhibitory effects against ciprofloxacin‐resistance bacteria; thus, ciprofloxacin resistance or tolerance may not affect the phage's infection ability. Additionally, researchers used phage‐ciprofloxacin combination therapy for the inhibition of multidrug‐resistant bacteria.

**Results:**

The sublethal concentrations of ciprofloxacin could lead to an increase in progeny production. Antibiotic treatments could enhance the release of progeny phages by shortening the lytic cycle and latent period. Thus, sublethal concentrations of antibiotics combined with phages can be used for the management of bacterial infections with high antibiotic resistance. In addition, combination therapy exerts various selection pressures that can mutually decrease phage and antibiotic resistance. Moreover, phage ciprofloxacin could significantly reduce bacterial counts in the biofilm community. Immediate usage of phages after the attachment of bacteria to the surface of the flow cells, before the development of micro‐colonies, could lead to the best effect of phage therapy against bacterial biofilm. Noteworthy, phage should be used before antibiotics usage because this condition may have allowed phage replication to occur first before ciprofloxacin interrupted the bacterial DNA replication process, thereby interfering with the activity of the phages. Furthermore, the phage‐ciprofloxacin combination showed a promising result for the management of *Pseudomonas aeruginosa* infections in mouse models. Nevertheless, low data are existing about the interaction between phages and ciprofloxacin in combination therapies, especially regarding the emergence of phage‐resistant mutants. Additionally, there is a challenging and important question of how the combined ciprofloxacin with phages can increase antibacterial functions. Therefore, more examinations are required to support the clinical usage of phage‐ciprofloxacin combination therapy.

## Graphical abstract

Bacteria resist phages through different possible mechanisms, such as inhibition of phage attachment to the bacterial receptors, inhibition of phage penetration into the bacterial cell, and cleavage of phage‐DNA after penetration to the bacterial cell. Additionally, bacterial biofilm is resistant to ciprofloxacin and the phage‐ciprofloxacin combination therapy can destroy the biofilm community of bacteria. To this end, phages could destroy the extracellular polysaccharide component of the biofilm matrix because of the presence of degrading tail enzymes. Phages can penetrate to the dipper layers of biofilm through the biofilm void spaces, and enhance the antibiotic activity. Finally, consecutively treatment of biofilms with phage and ciprofloxacin instead of a simultaneous application could lead to a better inhibitory effect against bacterial biofilm.

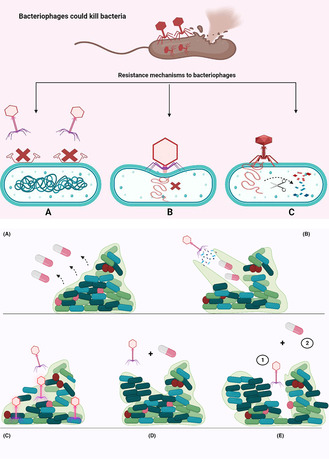

## 1 INTRODUCTION

Ciprofloxacin, a member of the fluoroquinolone drug class, is used to treat various Gram‐negative bacteria such as *Pseudomonas aeruginosa, Proteus mirabilis, Klebsiella pneumoniae*, and *Escherichia coli* and Gram‐positive bacteria such as *Staphylococcus aureus*. This antibiotic suppresses the activities of DNA‐gyrase and DNA topoisomerase during DNA replication, recombination, and repair.^1^ Ciprofloxacin resistance in different bacteria has been increasingly reported worldwide because of mutations in DNA gyrase genes (gyrA), efflux pumps, or plasmid‐mediated‐quinolone resistance in bacterial plasmids or chromosomes.^2^ In this regard, applying a combination of different substances such as nanoparticles, natural compounds, and bacteriophages with ciprofloxacin to enhance the efficacy of this antibiotic against multi‐drug resistant (MDR) bacteria has received much attention.^3^

Bacteriophages (phages) are viruses that could lyse bacteria while are not harmful to animal and human health. The resistance mechanisms to phages are different from antibiotics; therefore, phages have been widely used to treat MDR bacteria.^4^ Furthermore, phage‐antibiotic combination therapy could re‐sensitize resistant bacteria to antibiotics.^5^, ^6^

A phage cocktail, a mixture containing two or more phages with various host ranges in a single suspension, could kill bacteria more effectively.^7^, ^8^ Phage cocktails could lead to a better decrease in bacterial density and enhancement of the phages' activities.^9^ From this point of view, previous studies have shown that phage cocktail causes a higher reduction in bacterial infections.

Noteworthy, phages can destroy biofilm structure and improve the penetration of the antibiotic to the dipper layers of biofilm by inducing the synthesis of enzymes such as polysaccharide depolymerase.^10^ This enzyme can particularly destroy the macromolecule carbohydrates that exist in the envelope of the bacterial host and help the phage attach, penetrate, and lyse the bacterial cells.^11^ Additionally, phages could inhibit bacterial biofilm by inhibiting bacterial attachment, interference with quorum sensing, and the degradation of the exopolysaccharide matrix.^12^ Therefore, phages not only could kill bacteria but also could destroy the biofilm community of these microorganisms.

Although U.S. Food and Drug Administration has not yet approved, phages‐antibioticscombination therapy has been applied in different emergency clinical situations tomanage MDR infections.^13^ To this end, phage‐ciprofloxacin combination therapy is considered by scientists for enhancement of the ciprofloxacin efficacy, especially against MDR bacteria. In this review article, we will specifically discuss various aspects of this combination therapy to clarify the advantage and disadvantages of this combination therapy and promote its possible widespread use in the clinical setting.

## 2 THE USE OF PHAGES FOR CIPROFLOXACIN‐RESISTANT BACTERIA

As mentioned, resistance to ciprofloxacin is becoming an increasingly serious public health concern in the clinical setting. Recently published studies reported phages as a good therapeutic choice for the inhibition of ciprofloxacin‐resistant bacteria and in this section, we will evaluate these studies.

Ciprofloxacin is suggested for the treatment of salmonellosis; but, overuse of this antibiotic could lead to drug resistance, infection treatment failure, and severe clinical outcomes. Resistance to this antibiotic has been increasingly detected in different parts of the world.^14^ To this end, phages were used in different studies as an alternative approach for the inhibition of ciprofloxacin‐resistant *Salmonella*. *Pelyuntha* et al. isolated ciprofloxacin‐resistant *Salmonella* from the broiler production chain. Afterward, the authors isolated 11 *Salmonella* phages from wastewater samples derived from natural reservoirs, wastewater treatment stations, and broiler farms. The results of this study indicated that isolated phages could lyse (ranging from 33.3% to 93.3%) ciprofloxacin‐resistant bacteria. These data suggest that phages have a promising potential in combating ciprofloxacin‐resistant *Salmonella* isolates in the broiler production chain and preventing their spread through the food supply chain.^2^ In another study, a *Salmonella* Typhi isolates with decreased susceptibility to ciprofloxacin and producing CTX‐M‐15 extended spectrum‐lactamase (ESBL) was isolated in the Democratic Republic of the Congo. Different *Salmonella* phage clones (five and 14 batches of the commercial phage cocktail from INTESTI phage and Eliava R&D collection, respectively) were tested against isolated *S*. Typhi. Five phage clones from the Eliava collection indicated the ability to lyse the isolated *S*. Typhi without forming phage‐resistant mutants after 24 h of incubation, which is indicative of the potential of phages to suppress the growth of phage‐resistant mutants during phage therapy.^15^

**FIGURE 1:**
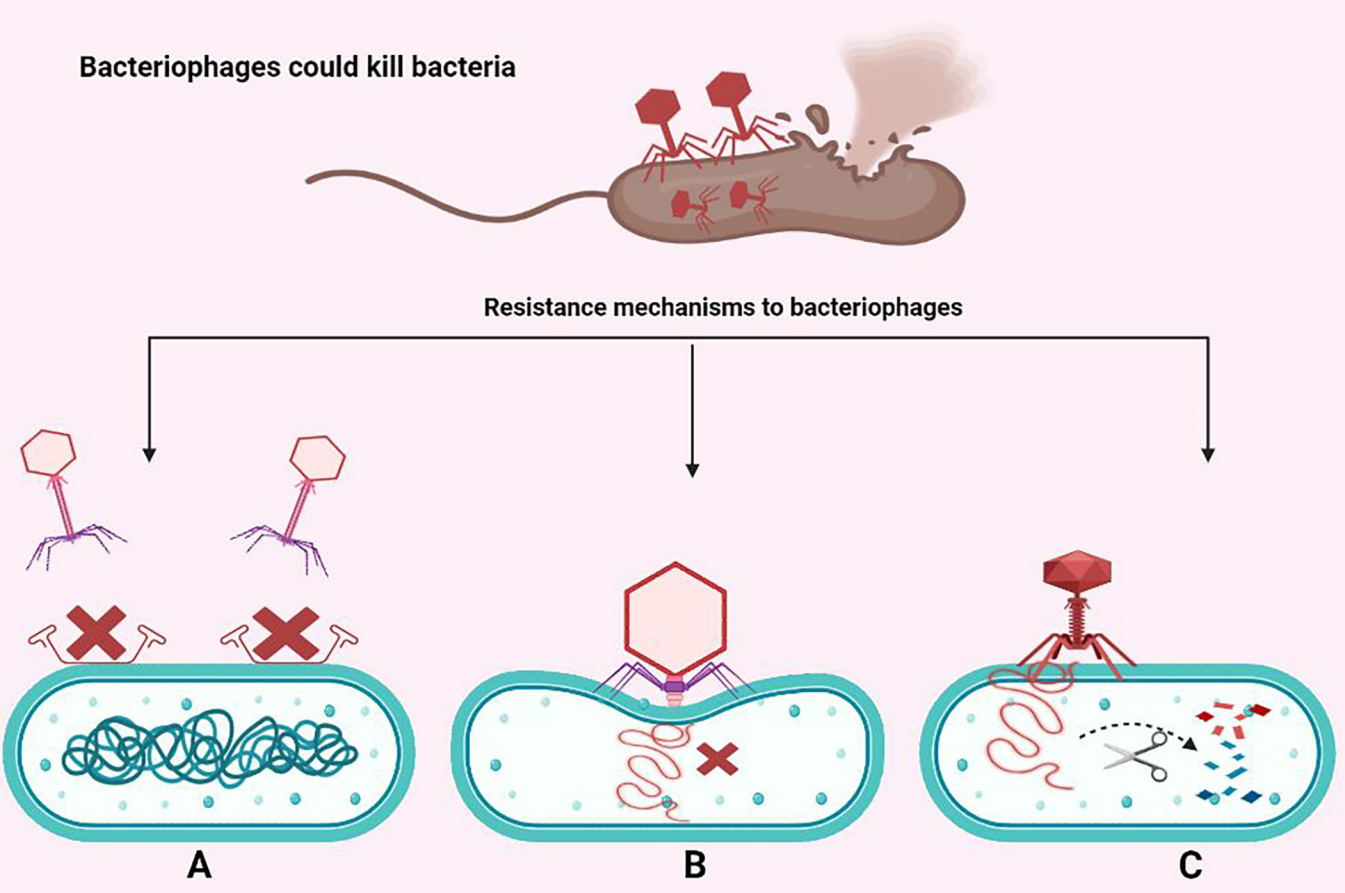
Resistance mechanisms of bacteria to phages. (A) Inhibition of phage attachment to the bacterial receptors. (B) Inhibition of phage penetration into the bacterial cell, and (C) cleavage of phage‐DNA after penetration to the bacterial cell.

These results supported *Jung* et al. data, who reported that phage P22 has a lytic activity against clinically isolated antibiotic‐resistant (levofloxacin, tetracycline, ciprofloxacin, and norfloxacin) *S*. Typhimurium and ciprofloxacin‐induced antibiotic‐resistant isolates of this bacterium. Noteworthy, this phage was obtained from Bacteriophage Bank at Hankuk University of Foreign Studies (Yongin, Gyeonggi, Korea). The P22 specificity to *S*. Typhimurium was not changed after the antibiotic resistance induction. Therefore, this phage could be applied as a biocontrol agent against *S*. Typhimurium. However, P22 showed the lowest lytic efficacy against the clinical isolates of this bacterium with antibiotic resistance.^16^ It's noteworthy to mention that bacteria could develop different mechanisms for phage resistance that could hinder the interactions between bacterial hosts and phages at multiple levels. These include mechanisms that prevent phage attachment to the bacterial surface, inhibition of phage‐DNA penetration into the bacterial cell, or phage‐DNA cleavage once inside the cell (Figure 1).^17^, ^18^ To this end, the specificity of phages against bacterial cells is mostly related to the binding affinity between receptor‐binding proteins in phages and receptors in the host. Hence, the alterations in receptors of the host cell surface are responsible for phage resistance, resulting in decreased lytic activity and this phenomenon should be considered in future studies.^19^, ^20^ Collectively, the recent WHO priority list of antibiotic‐resistant bacteria has shown fluoroquinolone‐resistant and ESBL‐producing *Salmonellae* as an important bacterium. The results of the mentioned studies would open the door for developing a new approach for managing ciprofloxacin‐resistant *Salmonella*. Nevertheless, to apply phages as a potential alternative agent against these bacteria, future studies are required to clarify the alterations in phage‐binding receptors in relation to the phage's lytic efficacy.

In addition to *Salmonella*, phages were also used for the inhibition of *S. aureus*. In this regard, the results of in vitro evaluation of a recent study showed that initial incubation of *S. aureus* with the minimum inhibitory concentrations (MICs) of commonly used antibiotics such as ciprofloxacin and mupirocin, could induce tolerance in this bacterium. Therefore, *S. aureus* tolerance to antibiotics might be easily induced clinically, for example when the uptake of orally administered antibiotics is hindered or when patients used antibiotics inappropriately for a long time. On the other hand, the authors reported that their Sa87 and Sa83 phages that were received from AmpliPhi Australia (Brookvale, NSW, Australia) lysed approximately 70% *S. aureus* clinical isolates and that the performance of phages was independent of antibiotic resistance profiles. Moreover, these phages destroyed the planktonic and biofilm community of antibiotic‐sensitive CI3 and ATCC51650 even after antibiotic tolerance was induced. Hence, the authors suggested that antibiotic resistance or tolerance does not affect the phages' infection ability.^21^ Therefore, recently published studies introduced phage therapy as a promising alternative treatment to manage MDR bacterial infections. However, unfortunately, the mentioned studies did not evaluate molecular interactions of phages and ciprofloxacin‐resistant bacteria; hence, this issue should be considered in future studies.

## 3 THE COMBINED USAGE OF PHAGES AND CIPROFLOXACIN

In the management of bacterial infections, the use of phages or antibiotics is restricted because of bacterial ability to develop resistance and the frequency of resistance is related to exposure to antibacterial agents. Two or more antibacterial agents, in combination therapy, using various mechanisms of action that could enhance each other's performance, decrease the chance of resistance evolution, and broaden activity spectra.^22^, ^23^ Phages have the specific ability to replication at the infection site, enhancing their density locally, at the expense of bacteria. However, bacteria by using different mechanisms could resist phages. Nevertheless, since the main mechanisms related to phage and antibiotics resistance do not overlap, the use of phage cocktails or phage‐antibiotics combination therapy has been recently suggested and evaluated to enhance the treatment's efficacy.^24^, ^25^, ^26^ In this regard, the researchers used different methods to study the inhibitory effects of phage‐ciprofloxacin combination therapy.

*P. aeruginosa*, a Gram‐negative bacillus, is naturally resistant to different antibiotic classes. Additionally, the continuous usage of antibiotics in patients with *P. aeruginosa* chronic infections, such as cystic fibrosis patients, could lead to additional antibiotic resistance. Mutations in *gyrA* or genes that are associated with the efflux pump expression can lead to resistance to different members of the fluoroquinolones family, such as ciprofloxacin.^27^, ^28^ The emergence of ciprofloxacin‐resistant in this bacterium is a serious challenge worldwide, bringing the need to find new approaches to manage this bacterium. To this end, phage–antibiotic combination was reported as a promising alternative to antibiotics for the inhibition of MDR *P. aeruginosa*.

*Ferran* et al. developed a creative use of the hollow fiber infection model (HFIM) to assess the potential benefit of phage‐ciprofloxacin combination therapy.^29^ The HFIM mimics concentration profiles observed in patients and is a preclinical closed system that allows the culturing of microbial cultures in an enclosed compartment. This compartment is usually a discrete cartridge that in turn is threaded with semi‐permeable fibers.^30^ The results of this study showed that each treatment selects for phage or antibiotic‐resistant clones in less than 30 h. On the other hand, the administration of ciprofloxacin after (4 h) the usage of phages, significantly removes the bacteria from the HFIM at 72 h. Phages‐ciprofloxacin combination therapy, based on the clinical regimens, suppressed the growth of resistant clones, providing opportunities to downscale the use of multiple antibiotics.^29^ In line with these results, another examination also reported that phage ELY‐1–ciprofloxacin combination suppressed *Escherichia coli* regrowth and caused less bacterial resistance than ciprofloxacin and phage used alone. Noteworthy, the usage of antibiotics in MIC and 6 h after phage therapy, showed the optimum inhibitory effect.^31^ Actually, a high amount of antibiotic may inhibit the DNA replication in bacterial cells, kill the host and prevent the replication of phages. This phenomenon may decrease the per‐host cell output of phage; thus, when the phages are added at a low MOI of 1, we do have not enough phage titer to significantly reduced bacterial cell count.^31^ Therefore, the decrease of the bacterial population size by the phages eradicates the minor population of spontaneous mutants less‐susceptible to ciprofloxacin that could thus not be selected afterward.

In another investigation also optical density‐based ‘lysis‐profile’ assays were used to evaluate the impact of colistin and ciprofloxacin on the bacteriolytic, bactericidal, and new‐virion‐production performance of three *P. aeruginosa* phages. Colistin significantly interferes with virion production and phage bacteriolytic function even at its MIC (1× MIC). Nevertheless, ciprofloxacin at 1× or 3 × MIC showed little inhibitory effect against phages. Therefore, the authors proposed ciprofloxacin as a promising antibacterial agent for combination therapy with phages especially when phage replication is required for treatment success.^32^

Furthermore, recently published studies also reported that the combination treatment of phages with ciprofloxacin significantly increases in susceptibility of MDR *P. aeruginosa* to antibiotics.^13^, ^33^, ^34^ In one of these studies, the presence of phage increased the number of *P. aeruginosa* strains susceptible to this antibiotic by 81%. Hence, the use of phage‐ciprofloxacin combination therapy, especially when we have MDR strains, could result in efficacious outcomes by re‐sensitizing the bacterial strain to the antibiotic.^13^ Furthermore, *Holger* et al. also reported that triple combination regimens including phage‐ciprofloxacin‐colistin lead to a remarkable reduction in MDR *P. aeruginosa* numbers. Besides, phage resistance was prevented or reduced in the presence of several classes of antibiotics.^34^

In addition to mentioned bacteria, the combination uses of phages and ciprofloxacin showed promising results for the inhibition of *S*. Typhimurium. The results of the study published in 2020 showed that the lytic activities of phage P22 were significantly increased in the presence of ciprofloxacin at pH 7 and the authors suggested that antibiotics play an important role in the host‐phage interaction, specifically in the adsorption process.^35^ Another study also reported that this phage combined with ciprofloxacin significantly suppressed the *S*. Typhimurium growth depending on the treatment order and time.^36^ The sub‐lethal concentrations of ciprofloxacin could lead to an increase in progeny production. Actually, antibiotic treatments could enhance the release of progeny phages by shortening the lytic cycle and latent period. Thus, combination therapy with phages and antibiotics (sub‐lethal concentrations) can be used for the management of infection caused by MDR bacteria.^3^ In addition, combination therapy exerts various selection pressures that can mutually decrease phage and antibiotic resistance. Furthermore, phages could block ciprofloxacin expulsion by modifying the efflux pump, leading to the accumulation of this antibiotic in *S*. Typhimurium.^23^ Finally, sub‐inhibitory concentrations of antibiotics could lead to the alteration in bacterial cell morphology which permits rapid phage maturation and accelerated cell lysis.^37^

Taken together, the combination usage of phages and ciprofloxacin is not only applicable but also synergistic in the decrease of bacterial populations and leads to the re‐sensitization of MDR bacteria to antibiotics. Nevertheless, low data are existing about the interaction process of ciprofloxacin and phages in combined therapies, especially regarding the emergence of phage‐resistant mutants and there is a challenging and important question of how the combined ciprofloxacin with phages can increase antimicrobial activity. For instance, the time of ciprofloxacin application and concentration of this antibiotic are essential factors that need to be considered in the combined treatment and should be evaluated in future studies.

It's noteworthy to mention that other studies also reported promising results for phage–ciprofloxacin combination in inhibition of different bacteria with huge antibiotic resistance such as *Burkholderia cepacia*, *Staphylococcus aureus*, and *Acinetobacter baumannii*. These studies are presented in Table 1.

**TABLE 1:** Studies used of phage‐ciprofloxacin combination for inhibition of different bacterial growth.

Year of publication	Bacteria	Phage	Model of study	Outcomes	References
2020	*P. aeruginosa*	vB_PaeP_4024 (*φ*24)	The Calu‐3 cell line and the isogenic CFTR knockdown cell line	Phage‐ciprofloxacin combination therapy remarkably maintained the integrity of the epithelial cell and suppressed the regrowth of bacteria.	[38]
2020	*A. baumannii*	vB_AbaP_AGC01	Human heat‐inactivated plasma blood model and an in vivo larva model	Phage‐ciprofloxacin or meropenem combination therapy showed synergistic activity.	[39]
2018	*S. Typhimurium*	P22	Agar diffusion test	The combination treatment effectively reduced the development of antibiotic resistance.	[40]
2018	*A. baumannii*	T4‐like bacteriophage KARL‐1	Liquid infection assays	Ciprofloxacin did generally not support phage activity.	[41]
2018	*P. aeruginosa*	PEV20	Time‐kill studies, and Air‐jet nebulizer	Ciprofloxacin indicated the most synergistic effect among the different phage‐antibiotic combinations therapy.	[42]
2017	*E. coli*	ECA2	Kill curves in Urine	Phage and ciprofloxacin combination therapy, at sub‐lethal concentration, reduced the bacterial counts in urine samples.	[43]
2016	*S. aureus*	SA11	Flow cytometric analysis and FIC	The sub‐lethal concentration of ciprofloxacin injured bacterial cells, but phage and ciprofloxacin combination therapy showed a better inhibitory effect.	[44]
2016	*S. aureus*	ɸAPCEc03, ɸAPCEc02, and ɸAPCEc01	A miniaturized enumeration method	The simultaneous usage of three phages as a cocktail entirely inhibited and prevented the emergence of resistant mutants.	[45]
2015	*Burkholderia cenocepacia*	KS12 and KS14	Double‐layer agar assay	When grown in sub‐inhibitory concentrations of ciprofloxacin, cells developed an elongated morphology.	[37]
2022	*E. coli*	Endolysin EC340 obtained from phage PBEC131	Checkerboard Assay	An additive effect was seen with ciprofloxacin.	[46]
2021	*E. coli*	HK97	Double agar overlay assays	The use of the correct sub‐lethal concentration of antibiotics in combination with phages could completely eradicate bacteria.	[47]
2020	*P. aeruginosa*	Not reported	Nonlinear population dynamics model.	Combination therapy showed a better inhibitory effect in comparison to the phage or antibiotic alone and enhanced the performance in interaction with the innate immune system.	[48]

Abbreviation: FIC, Fractional inhibitory concentration.

## 4 THE PHAGE‐CIPROFLOXACIN COMBINATION THERAPY FOR INHIBITION OF BACTERIAL BIOFILM

Different bacteria, especially *P. aeruginosa* and methicillin‐resistant *S. aureus* (MRSA), after attachment to various surfaces become embedded in self‐secreted molecules, such as proteins polysaccharides, and extracellular DNA. These molecules are associated with the structural integrity of the biofilm and the biofilm matrix. The biofilm community can act as a barrier to physical, chemical, and biological challenges. Moreover, this structure can tolerate up to 1000 times higher antibiotic concentrations than planktonic cells due to the lack of antibiotic penetration into the complex polysaccharide matrix (glycocalyx) of biofilms.^4^ Thus, because of the determinant role of bacterial biofilm in the development of chronic and drug‐resistant infections, a new antibacterial approach is urgently required for the management of chronic infections caused by persistent biofilms. To this end, the use of phages was reported by researchers as a promising approach for the enhancement of ciprofloxacin activity against bacterial biofilm.

In a study published in 2020, six *P. aeruginosa* isolates were collected from wound patients and cystic fibrosis then, combinations of ciprofloxacin and PEV20 (anti‐*Pseudomonas* phage) were used for the destruction of the biofilm community of bacteria. Treatment with ciprofloxacin (at MIC) or phage alone could not decrease the biofilm viability; however, combination therapy significantly reduced biofilm and facilitated biofilm disruption and removal.^25^ The authors proposed that the air‐biofilm interface of ciprofloxacin could lead to bacterial filamentation in the biofilm community while those residing in the interior were spared. A low amount of oxygen and reduced bacterial metabolic activity in the mid‐layer of the biofilm could cause antibiotic tolerance. On the other hand, the integrity of the extracellular matrix could be damaged by phages, consequently, exposing the metabolically inactive bacteria to surrounding nutrients in the media. Subsequently, both phages and ciprofloxacin could suppress bacteria when bacteria become metabolically active.^25^, ^49^ Additionally, phages can diffuse and penetrate the dipper layers of the biofilm, amplify and remain viable within the complex biofilm matrix. Actually, the high density of bacteria in the biofilm community is favorable for the phages to multiply, resulting in high local titers and the rapid spread of phage infections.^25^, ^50^, ^51^ Noteworthy, PEV20‐tobramycin combination did not show a synergistic antimicrobial effect against 48‐h biofilm in all seven strains. Fluoroquinolones, such as ciprofloxacin, more effectively penetrate biofilm in comparison to tobramycin and other aminoglycosides.^25^ These findings were in line with *Issa* et al. results, which reported that co‐administration of ciprofloxacin and phages work synergistically to affect biofilms.^52^ Therefore, the authors suggested ciprofloxacin as an applicable antibacterial agent for combination therapy with phages against *P. aeruginosa* biofilm.

**FIGURE 2:**
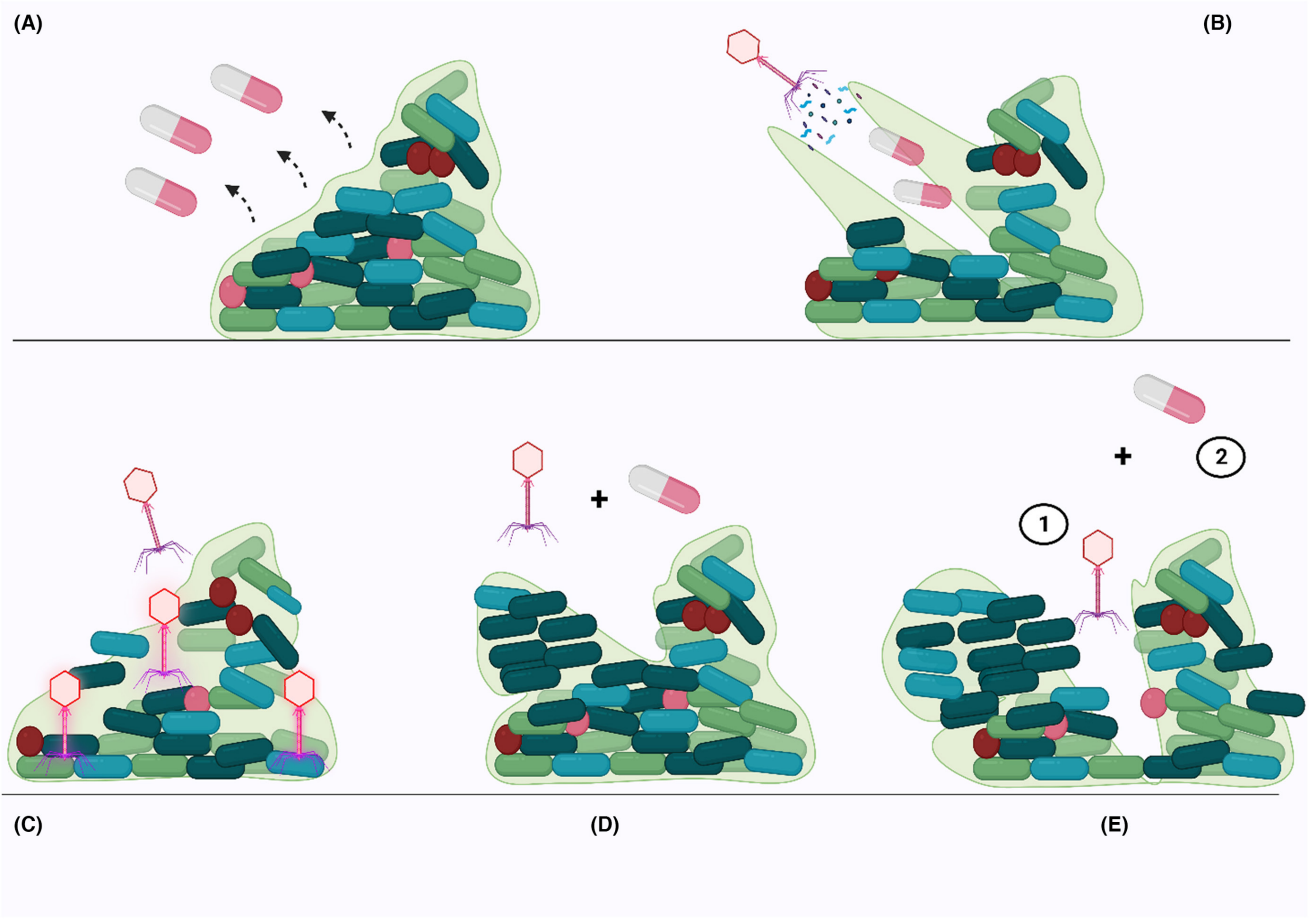
The phage‐ciprofloxacin combination therapy against bacterial biofilm. (A) Bacterial biofilm is resistant to ciprofloxacin. (B) Phages could destroy the extracellular polysaccharide component of the biofilm matrix because of the presence of degrading tail enzymes. (C) The penetration of phages to dipper layers of biofilm through the biofilm void spaces. (D) and (E) consecutively treatment of biofilms with phage and ciprofloxacin instead of a simultaneous application, could lead to a better inhibitory effect against bacterial biofilm.

Furthermore, the findings of another experiment demonstrated that phages have the optimum effect against bacterial biofilm in the beginning steps of biofilm formation. In this regard, treatment with phages was less effective against 72‐h flow‐cell biofilms and was most effective against young (24 h) biofilm communities; therefore, the large bacterial micro‐colonies seemed to be a defense mechanism against phage attack.^53^ Phage‐ciprofloxacin combination significantly removed biofilm and the phage application before the antibiotic had the best effect on biofilm treatment. This effect could be due to the high amount of bacterial cells in that the phages can multiply, compared to conditions when the phages are added after antibiotic therapy.^53^ In line with these results, in the recently published study, the authors formed 48 h *P. aeruginosa* biofilm, then used *P. aeruginosa* infecting phage and different antibiotics such as ciprofloxacin, alone and in sequential or simultaneous combinations. Antibiotics or phage alone showed a modest inhibitory effect against bacterial biofilm and a significant enhancement in the inhibitory effect was detected when these antibacterial agents were used simultaneously. To this end, the administration of ciprofloxacin after 6 h of phage treatment leads a profound reduction, below the detection limit, in the biofilm community (Figure 2).^54^

In addition, another investigation also reported that simultaneous usage of the phage‐antibiotic combination therapy did not indicate synergism in the case of ciprofloxacin, but show synergistic activity in the case of fosfomycin.^55^ To this end, the authors proposed ciprofloxacin by interference in the replication of DNA in the host cells has more effects on phage activities in comparison to fosfomycin which suppresses the synthesis of the cell wall. Actually, recently published studies that evaluated biofilm structures reported that consecutively of ciprofloxacin and phages for inhibition of bacterial biofilm could lead to synergistic activity, while simultaneous usage of these agents may cause antagonism. Noteworthy, the use of ciprofloxacin after the administration of phages has allowed phage replication to occur first before ciprofloxacin interrupted the bacterial DNA replication process, thereby interfering with the activity of the phages.^23^, ^54^, ^55^ *Tkhilaishvili* et al. also support these findings but this time for MRSA biofilm. These authors reported that pre‐exposure to phage, Sb‐1, followed by ciprofloxacin treatment, destroyed the MRSA biofilm with even lower antibiotic concentrations. Actually, simultaneous usage of phage and antibiotic did not show an inhibitory effect against MRSA biofilm, while pretreatment with phage remarkably increased the bactericidal activity of the antibiotic.^56^

Therefore, based on the mentioned findings, immediate usage of phages after the attachment of bacteria to the surface of the flow cells, before the development of micro‐colonies, could lead to the best effect of phage therapy against *P. aeruginosa* biofilm. Additionally, phage application should precede antibiotic treatment; however, more studies are required to prove these results.

It should be noted that microorganisms with different antibiotic‐resistance characteristics interact with each other in a biofilm community. Therefore, treating an infection caused by a multi‐species biofilm is more challenging than that of a one‐species biofilm. On this point, recent studies have assessed the inhibitory effect of phage‐antibiotic staggered treatment against mixed‐species biofilms.^57^ For instance, an interesting study that was published in 2019 evaluated the in vitro effect of either the staggered or simultaneous usage of ciprofloxacin and phages against *P. aeruginosa/S. aureus* dual‐species biofilms. The combination of phages and ciprofloxacin indicated a significant enhancement in the inhibitory effect of both antibacterial agents with complete eradication of dual‐species biofilms after staggered exposure to Pyophage or Pyophage + Sb‐1 (Staphylococcal phage) for 12 h followed by 1 mg/L of ciprofloxacin. Actually, the highest anti‐biofilm performance could be reached when ciprofloxacin was added after 6 or 12 h of pre‐exposure to PYO + Sb‐1. Pre‐incubation of dual‐species biofilms with phages for 3 or 24 h prior addition of ciprofloxacin showed higher minimum biofilm eradicating concentration values, thus, not only dosage but also an optimal time of antimicrobial exposure is essential in the usage of combination therapy.^58^

Roszak and co‐authors also evaluated the effectiveness of two phages, vB_SauM‐D and vB_SauM‐A, combined with ciprofloxacin against dual‐species biofilm community consisting of *S. aureus* and *Candida albicans*. The presence of *C. albicans* decreased bacterial eradication and a reduction of biofilm‐specific activity of phage‐antibiotic combination therapy. Usage of ciprofloxacin (1 mg/L) and phages caused a 90% and 69% reduction of biofilm‐specific activity of mono and dual‐species biofilm community, respectively.^59^ Therefore, as mentioned in the above studies, phage‐ciprofloxacin combination therapy should be considered for the management of polymicrobial biofilm infection. These infections are less common, but their treatment presents a major challenge little has been published about polymicrobial biofilm infections; therefore, more in vitro and clinical evaluations are required to support the development of phage/antibiotic combination therapy for polymicrobial biofilm.

Finally, it's noteworthy to mention that the microscopic evaluation of *Tkhilaishvili* and co‐authors indicated that phages could destroy the extracellular polysaccharide component of the biofilm matrix because of the presence of degrading tail enzymes. This condition facility the penetration of antibiotics to the dapper layer of the biofilm.^56^ Thus, phages could destroy the matrix of biofilm by producing an enzyme or induce enzyme production by the bacterial host, consequently enhancing the aeration of the interiors of biofilm, a fusion of ciprofloxacin, and the spread of phages.^60^ In this regard, *Verma* et al. indicated depolymerase/ciprofloxacin combination treatment *K. pneumoniae biofilm* (5 days old); however, the significant improvement was only detected when pretreatment with depolymerase was followed by ciprofloxacin administration. These authors also reported a similar potential for two phages, non‐producing and producing depolymerase, in inhibition of the planktonic community of *K. pneumoniae*. On the other hand, phage non‐producing depolymerase, contrary to depolymerase producer, was not able to decrease the sessile colony count.^61^ In line with this study, in a recently published study, different antibiofilm agents such as ciprofloxacin, lytic phages (non‐producing and producing depolymerase), and recombinant phage‐encoded enzyme were used for inhibition of MDR *K. pneumonia* biofilm. The authors reported depolymerase‐producing phage with ciprofloxacin as the most effective antibiofilm combination against bacterial biofilm.^62^

Thus, depolymerase originating from phage can be used as a supportive antibiofilm agent for ciprofloxacin; however, depolymerase was not detected in some phages with antibiofilm activity. For instance, *Akturk* et al. reported that this enzyme was not identified in the EPA1 genome, a lytic *Pseudomonas* phage that was isolated from hospital sewage and belongs to the *Myoviridae* family. Therefore, the authors reported that they have no evidence that phage depolymerase is responsible for the synergistic action of the phage‐antibiotic combination therapy against bacterial biofilm. Indeed, the authors proposed that the penetration of phages to inner layers of biofilm through the biofilm void spaces should be considered. Because after this phenomenon, phages could replicate in dipper layers of biofilm, reach high titers, and interrupt the biofilm matrix.^54^ Taken together, although different studies have reported depolymerase as an essential factor for the inhibition of bacterial biofilm, more confirmatory studies are required.

Other studies that used phage‐ciprofloxacin staggered treatment for inhibition of bacterial biofilm are presented in Table 2.

**TABLE 2:** Studies used of phage‐ciprofloxacin combination for reduction of bacterial biofilm.

Year of publication	Bacteria	Phage	Model of study	Outcomes	References
2022	STEC	ECML‐117 and vB_Eco4‐M7	Different methods	The phage cocktail‐ciprofloxacin or‐rifampin combination therapy showed a synergistic effect and effectively suppressed STEC biofilm community. Ciprofloxacin was almost as efficient in inducing prophages from the bacteria.	[63]
2016	*S. aureus*	Phage SA11 (PB Number: BP 6002)	Disk diffusion, Time‐kill curve analysis, and Congo red agar assay.	Combination therapy leads to a remarkable reduction in biofilm formation.	[64]
2014	*S. aureus*	Endolysin LysH5	Different methods	LysH5 showed activity against biofilm and persister cells obtained after treatment with ciprofloxacin.	[65]
2011	*Streptococcus suis*	A bacteriophage lysin (designated LySMP)	Twenty‐four‐well plate biofilm assay, EPS assay	This phage lysin in combination with antibiotics synergistically inactivated the released cells and increased the dispersal of bacterial biofilm.	[66]
2010	*K. pneumoniae*	Depolymerase‐producing lytic bacteriophage (KPO1K2)	Different methods	Combination therapy more efficiently reduced bacterial numbers of older biofilm in comparison to individual therapy. Ciprofloxacin alone could not significantly decrease the bacterial count in established biofilms.	[61]
2009	*K. pneumoniae*	T7‐like lytic bacteriophage KPO1K2	Different methods	In comparison to the individual treatment, combination therapy significantly killed bacteria and prevented the formation of resistant variants.	[60]
2020	*E. coli*	ɸWL‐3	LB broth containing porous glass beads	The co‐administration of phage with antibiotics, especially after staggered exposure, enhanced the performance of antibiotics against biofilm.	[55]

Abbreviations: EPS, Exopolysaccharide, STEC, Shiga toxin‐producing *E. coli*.

## 5 IN VIVO USAGE OF PHAGE‐CIPROFLOXACIN COMBINATION

As fully discussed in the previous sections, the combination use of ciprofloxacin and phages to manage MDR bacteria and biofilm community has been reported in many in vitro studies; nonetheless, this combination therapy has not been well received in clinical andin vivo research. However, in this section, we will review in vivo and clinical experiments that have used phage‐antibiotic staggered treatment for the management of bacterial infections.

In this regard, different studies used inhalable powder by co‐spray drying *Pseudomonas* phage PEV20 with ciprofloxacin. In one of these studies, lung infection was established in neutropenic mice then these animals were treated with ciprofloxacin, phage, and PEV20‐ciprofloxacin treatment using a dry powder insufflator. PEV20‐ciprofloxacin combination powder remarkably decreased inflammation and the load of MDR *P. aeruginosa* in mouse lungs, while no obvious reduction in the bacterial load was observed when the animals were treated only with ciprofloxacin or PEV20.^67^ The findings of *Chan* et al. also demonstrated that PEV20 and ciprofloxacin‐PEV20 powders remained stable over long‐term storage and showed remarkable antibacterial activities against lung mouse lung infection caused by *P. aeruginosa*.^68^ Other studies also reported that ciprofloxacin can sufficiently stabilize phage through verification and/or hydrogen bonding at 4°C and co‐spray dried phage PEV20‐ciprofloxacin combination dry powder formulations could lead to the synergistic antibacterial effect against MDR *P. aeruginosa* isolates.^69^, ^70^ In addition to lung infection, rats with aortic experimental endocarditis were treated with an anti‐*Pseudomonas* phage cocktail alone or combined with ciprofloxacin. The results showed that phage/ciprofloxacin combinations were highly synergistic, successfully managed 64% (7/11) of rats, and suppressed the regrowth of phage‐resistant mutants.^71^

In another interesting study, the authors reported an 88‐year‐old man with a relapsing *P.aeruginosa* prosthetic knee infection. The phages (a cocktail of three phages) were used for the treatment of this patient because prosthesis explantation and/or exchange was not feasible. In this regard, phage suspension was injected after conventional arthroscopy, then the patient was treated with a suppressive antimicrobial therapy including intravenous ceftazidime and then oral ciprofloxacin. The mentioned treatment regimen rapidly manages the infection with the disappearance of signs of pain in the left knee and heart failure. To this end, the authors proposed that phage therapy has the potential to be used as salvage therapy for the treatment of relapsing prosthetic joint infection caused by *P. aeruginosa*, in combination with suppressive antimicrobial therapy.^72^

Therefore, the phage‐ciprofloxacin combination showed a promising result for the management of *P. aeruginosa* infections in mouse models. Although the mechanisms underlying synergy are yet to be elucidated, it seems the modified antibacterial function of phage‐ciprofloxacin combination therapy is due to selective pressure under which the bacteria mutate in one trait to improve fitness while suffering a decrease in another trait. Indeed, by acquiring phage resistance via the loss of phage‐binding receptor, the bacteria regained sensitivity to several classes of antibiotics including ciprofloxacin.^23^, ^67^, ^73^ Nevertheless, the exact function of this combination therapy should be evaluated in future studies and more in vivo evaluations could lead to further insights and support the development of phage‐ciprofloxacin combination therapy.

## 6 CONCLUSION

Different bacteria have been shown reduced susceptibility and resistance to ciprofloxacin. To this end, phages and their derivatives were reported as an alternative strategy for the enhancement of ciprofloxacin efficacy even for bacteria with high resistance to this antibiotic. Additionally, combination therapy of phages and ciprofloxacin showed a promising inhibitory effect against the biofilm community of different bacteria, especially *P. aeruginosa*. Animal studies also provided a proof of concept to use the phage‐ciprofloxacin combination to concomitantly treat MDR infections. However, the exact interaction of ciprofloxacin and phages have been not elucidated yet, and the data from in vivo studies are very limited. Thus, more and more animal and in vivo evaluations are needed to evaluate molecular interactions of phages with ciprofloxacin; additionally, pharmacokinetics and chronic safety of phage‐ciprofloxacin staggered treatment should be considered in future studies.

## AUTHOR CONTRIBUTIONS

AS, MN, and ZC conceived and designed the study, contributed to comprehensive research, and wrote the manuscript. Notably, all authors have read and approved the manuscript.

## CONFLICT OF INTEREST STATEMENT

The authors declare that the research was conducted in the absence of any commercial or financial relationships that could be construed as a potential conflict of interest.

## Data Availability

The authors confirm that the data supporting the findings of this study is available within the article.
